# Disrespect and abuse of women during the process of childbirth at health facilities in sub-Saharan Africa: a systematic review and meta-analysis

**DOI:** 10.1186/s12914-020-00242-y

**Published:** 2020-09-07

**Authors:** Zemenu Yohannes Kassa, Berhan Tsegaye, Abebaw Abeje

**Affiliations:** Department of Midwifery, College of Medicine and Health Sciences, Hawassa University, Hawassa, Ethiopia

**Keywords:** Meta-analysis, Childbirth, Disrespect, Abuse, Sub-Saharan Africa

## Abstract

**Background:**

Disrespectful and abusive treatment of women by health care providers during the process of childbirth at health facility is an international problem. There is a lack of data on disrespect and abuse of women during the process of childbirth at health facilities in Sub-Saharan Africa. The purpose of this study was to determine the prevalence of disrespect and abuse of women during the process of childbirth at health facilities in sub-Saharan Africa.

**Methods:**

The PRISMA guideline protocol was followed to write the systematic review and meta-analysis. Published studies were searched from Medline, PubMed, CINAHL, EMBASE, Maternal and infant care, science direct, and PsycINFO. Articles were accessed by three reviewers (ZY, BT and AA) using the following key terms, “attitude of health personnel” AND “delivery obstetrics*/nursing” OR “maternity care” AND “disrespect” OR “abuse” OR “professional misconduct” AND “parturition” AND “prevalence” AND “professional-patient relations” AND “Sub-Saharan Africa”. Additional articles were retrieved by cross referencing of reference. The heterogeneity of studies were weighed using Cochran’s Q test and I^2^ test statistics. Publication bias was assessed by Egger’s test.

**Results:**

Thirty three studies met the inclusion and included in this systematic review and meta–analysis of disrespect and abuse of women during the process of childbirth at health facilities. The pooled prevalence of disrespect and abuse women during the process of childbirth at health facilities in Sub-Saharan Africa was 44.09% (95% CI: 29.94–58.24).Particularly physical abuse was 15.77% (95% CI: 13.38–18.15), non-confidential care was 16.87% (95% CI: 14.49–19.24), abandonment was 16.86% (95% CI: 13.88–19.84) and detention was 4.81% (95% CI: 3.96–5.67).

**Conclusion:**

In this study disrespect and abuse of women during the process of childbirth at health facilities are high compared with other studies, particularly non-confidential care and abandonment his high compared with other studies. This study points out that the ministry of health, health care providers, maternal health experts shall due attention to women’s right during the process of childbirth at health facilities.

## Background

Disrespectful and abusive behaviors on woman during the process of child birth at health facilities is a public health concern, which violates woman dignity, integrity, and respectful care in maternity units [1]. The Mistreatment of a woman during the process of child birth at health facilities has become an international agenda by maternal and child health advocators [2]. It is a violation of the fundamental human rights of women, newborns, and families [3]. Disrespect and abuse of women during the process of childbirth at health facilities is a violation of women’s rights, health, self-determination, privacy, bodily integrity, family life, freedom from discrimination, and spiritual freedom [4].

Every woman has the right to get quality of health care which is respectful, dignified, free of violence, free of discrimination, the right to know the procedure and any activities related to health care [5], nevertheless disrespect, abuse and abandonment of women during the process of childbirth at health facilities constitute seriously violation of women rights, which acknowledged across in the world [6, 7].

Various form of disrespect and abuse of women during the process of childbirth at health facilities have been stated in the literature such as; non-consented care, non-confidential care, non-dignified care, physical abuse, discrimination based on specific attributes, abandonment or denial care and detention in the health facilities due to inability to pay medical expense [8, 9].

Importantly, disrespect and abuse during child birth is any act of in the following lists physical abuse (use of force and physical restraint), sexual abuse, verbal abuse (harsh language, threats and blaming), stigma and discrimination (discrimination based on sociodemographic characteristics, and discrimination based on medical conditions), failure to meet professional standards of care (lack of informed consent and confidentiality, physical examinations and procedures, neglect and abandonment), poor rapport between women and providers (ineffective communication, lack of supportive care, loss of autonomy), and health systems conditions and constraints (lack of resources, lack of policies, facility culture) [10].

In addition to the health care providers might be made physical violence like punching, slapping, pushing, beating, poking, forced examination (abdominal and vaginal examination without consent), excessive and inappropriate medical interventions, episiotomy and stitching without anesthesia during childbirth [9]. Furthermore, a study conducted in India showed that 9.1% of experienced disrespect and abuse by self reporting, whereas observers reported 22.4% of women being mistreated [11]. Similarly, a study conducted in India showed that 71.3% experienced disrespect and abuse [12].

Pregnancy and childbirth are momentous events, which lives in women and families in every community in the world. Whereas, woman’s positive or negative experience during childbirth stays with her throughout her lifetime [13].

Despite the last two decades remarkable achievements have made on maternal and child health in the world, still there is a large number of maternal and neonatal mortality across the globe. Mistreatment and obstetric violence is a powerful deterrent of women to seek care in health facilities for their subsequent deliveries [14, 15]. Stakeholders and concerned bodies to achieve Sustainable Development Goal year 2030, respectful maternity care takes a pivotal for women’s utilization of maternity care services. One of the key strategies to lessen maternal and neonatal mortality is increasing institutional delivery, and woman friendly care.

There is a lack of studies on the disrespect and abuse of women during the process of childbirth at health facilities in Sub-Saharan Africa. This study gave a piece of information on the status of disrespect and abuse of women during the process of childbirth at health facilities for obstetric care providers, policy planners, the ministry of health and relevant stakeholders for possible mitigation of disrespect and abuse of women during the process of childbirth at health facilities. Therefore, the aim of this study was to quantify the prevalence of disrespect and abuse of women during the process of childbirth at health facilities in sub-Saharan Africa.

## Method

### Search strategies

This systematic review and meta-analysis were done based on published studies. The search strategy included the following data bases: PubMed/Medline, CINAHL, EMBASE, Maternal and infant care, science direct, and PsycINFO were systematically searched. The search was carried out from January 06 to June 04, 2020. Articles were retrieved by three reviewers (ZY, BT and AA) using MeSH terms, “attitude of health personnel” AND “delivery obstetrics*/nursing” OR “maternity care” AND “disrespect” OR “abuse” OR “professional misconduct” AND “parturition” AND “prevalence” AND “professional-patient relations” AND “Sub-Saharan Africa”. Additional articles were retrieved by using cross referencing of references, titles and abstracts.

### Eligibility criteria

Studies reported all age women who experienced disrespect and abuse during the process of childbirth at health facilities in sub-Saharan Africa were eligible for this review. Quantitative primary studies were conducted with cross sectional, and cohort study design in sub-Saharan Africa, irrespective of whether the study was implemented in the health facility and or in the community were included. Limit of the language is the English and 2000–2020 published were included.

### Exclusion criteria

Studies which are, qualitative studies, review studies, conference abstract, articles incomplete information, with methodological problems or with full text not available were excluded.

### Data screening and extraction

Three authors (ZY, BT, and AA) independently extracted all necessary data using a standardized data extraction format. Three reviewers ((ZY, BT, and AA) independently extracted the data from eligible articles using the Joana Brigg’s Institute (JBI) critical appraisal checklist for simple prevalence which contain nine checklist items [16]. Discrepancies during scoring were resolved through discussion and consensus by reviewers. Three reviewers independently evaluated and cross checked the score, and the articles weighed > 4.5 points were considered as high quality score (Table S1).

### Data analysis

Data analysis was implemented using Stata statistical software, version 15 (StataCorp LP, College Station, TX, USA). The pooled prevalence of disrespect and abuse of women during the process of childbirth at health facilities with 95%CI was calculated using the random effects model, due to the possibility of heterogeneity among studies. The heterogeneity test was assessed by using the *I*^2^ statistics and Q statistics test. The publication bias was assessed using the Egger‘s regression test objectively and funnel plot subjectively. Any asymmetry of a funnel plot and statistical significance of Egger’s regression test (*P*-value < 0.05) was suggestive of publication bias [17, 18].

**Fig. 1:**
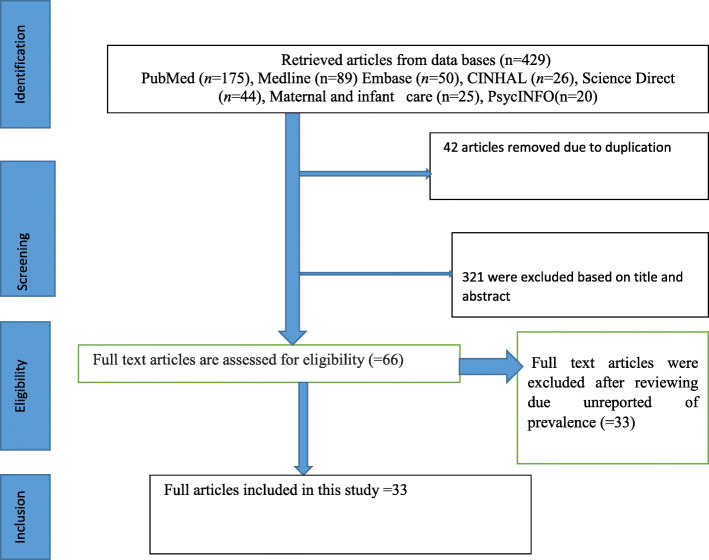
PRISMA Flow diagram

Preferred Reporting Items for Systematic Reviews and Meta-Analyses (PRISMA) guidelines was strictly followed during the systematic review and meta-analysis [19]. Four hundred twenty nine articles were accessed, from this 42 articles excluded due to duplication. Three hundred thirty three were excluded based on title and abstract. The rest of 66 articles were reviewed full articles. Thirty three articles were excluded after full article reviewing due to qualitative study (unreported prevalence). Finally, 33 studies were included in this systematic review and meta-analysis (Fig. 1). The heterogeneity test showed that I^2^ = 99.9%, *p*-value is 0.000 and publication bias (Egger’s test p- value is 0.34).

## Result

In this systematic and meta-analysis, a total of 21,330 population has participated, 3686 population has participated in community based studies, 16,224 population was laboring mothers and 1420 population was health care providers. The sample size of study population varied from 54 to 2109 (Table 1).

**Table 1:** Prevalence of disrespect and abuse during childbirth and maternity care in Sub-Saharan Africa: a systematic review and meta-analysis [20–47]

Author	Year of Pub.	Country	Study population	Sample size	Case	Over all Pre. (%)	Physical abuse (%)	Non confidential (%)	Detention (%)	Abandonment (%)	Quality
Asefa and Bekele [20]	2015	Ethiopia	Laboring mothers	173	136	78.6	32.9	21.4	0.6	39.3	5
Anteneh et al. [21]	2018	Ethiopia	Providers	54	14	25.9	25.9	34.5	18	13.2	5
Sheferaw et al. [22]	2017	Ethiopia	Laboring mothers	240	87	36.0	9.0	17.0	NR	19.0	6
Wasihun B et al. [23]	2018	Ethiopia	Community	410	275	67.1	57.6	11.0	NR	7.1	7
Wassihun and Zeleke [24]	2018	Ethiopia	Laboring mothers	284	121	42.6	34.5	31.7	NR	32.4	6
Kathleen P et al. [25]	2018	Ethiopia	Laboring mothers	204	43	21.1	0.5	13.7	0	2.5	6
Gebremichael et al. [26]	2018	Ethiopia	Community	1125	248	22.0	0.8	0.8	3.8	6.0	8
Ukke et al. [27]	2019	Ethiopia	Laboring mothers	281	278	98.9	29.5	17.1	NR	4.3	5
Mihret [28]	2019	Ethiopia	Laboring mothers	409	307	75.1	46.9	32.3	NR	12.7	6
Bobo et al. [29]	2019	Ethiopia	Laboring mothers	612	458	74.8	37.1	40.4	2.9	25.2	7
Siraj et al. [30]	2019	Ethiopia	Laboring mothers	290	266	91.7	87.9	50	25.9	53.8	6
Bekele [31]	2020	Ethiopia	Community	316	247	78.2	21.5	33.9	0.3	13.3	7
Abuya T et al. [32]	2015	Kenya	Laboring mothers	641	129	20.1	4.2	8.5	8.1	14.3	7
Atai et al. [33]	2018	Kenya	Laboring mothers	164	53	32	1	28	NR	22	5
Sando et al et al. [34] H	2014	Tanzania	Laboring mothers	147	18	12.2	2.7	0.7	0.7	6.8	5
Sando et al et al. [34] N	2014	Tanzania	Laboring mothers	1807	271	15.0	4.7	1.8	0.1	7.9	8
Kruk et al. [35] L	2014	Tanzania	Laboring mothers	1779	343	19.28	2.9	4.39	0.17	8.53	9
Kruk et al. [35] C	2014	Tanzania	Community	593	167	28.16	5.08	6.16	0.34	15.54	8
Kujawski S et al. [36]	2015	Tanzania	Laboring mothers	1388	247	17.79	NR	NR	NR	NR	9
Sando et al. [37] L	2016	Tanzania	Laboring mothers	1914	278	14.5	5.0	2.0	0.2	8.0	5
Sando et al. [37] C	2016	Tanzania	community	64	50	78.0	52.0	54.0	2.0	52.0	8
Kujawski SA et al. [38]	2017	Tanzania	Laboring mothers	644	84	13.1	2.5	1.74	2.37	6.09	9
Freedman et al. [39] P	2018	Tanzania	providers	232	162	69.8	13.79	10.78	5.17	18.1	6
Freedman et al. [39] L	2018	Tanzania	Laboring mothers	232	23	9.91	0.86	1.29	1.29	3.45	6
Larson et al. [40]	2018	Tanzania	Laboring mothers	2002	286	14.3	NR	NR	NR	NR	5
Bishanga. et al. [41]	2019	Tanzania	Community	732	535	73.1	4.6	32.9	30.9	16.7	7
Galle et al. [42] R	2019	Mozambique	Laboring mothers	302	73	24	0	NR	0.3	7.3	5
Galle et al. [42] U	2019	Mozambique	Laboring mothers	218	175	80	0	NR	0	34.9	5
Sethi et al. [43]	2017	Malawi	Laboring mothers	2109	41	1.9	NR	0.2	58.2	10	8
Okafor et al. [44]	2015	Nigeria	Community	446	437	98.0	35.7	26.0	22.0	29.1	7
Ijadunola et al. [45]	2019	Nigeria	Laboring mothers	384	73	19.1	1.6	5.2	0.5	6	5
Moyer et al. [46]	2016	Ghana	providers	853	614	72.0	NR	NR	37.9	NR	6
Wesson et al. [47]	2018	Namibia	Providers	281	88	31.0	30	NR	NR	NR	5

*H* HIV positive, *N* HIV negative, *P* Health care providers, *C* Community, *L* Laboring mother, *CS* Cross-sectional study

### Meta-analysis

**Fig. 2:**
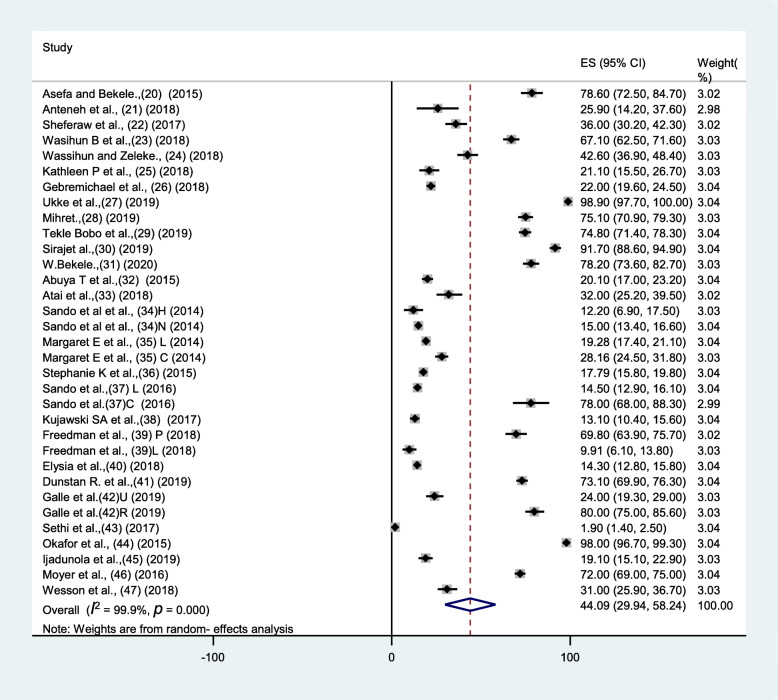
The forest plot Prevalence of disrespect and abuse during childbirth and maternity care in Sub-Saharan Africa

The prevalence of disrespect and abuse of women during the process of childbirth at health facilities in Sub-Saharan African was 44.09% (95% CI: 29.94–58.24).The I^2^ statistic for disrespect and abuse of women during the process of childbirth at health facility was 99.94% (Fig. 2).Subgroup analysis was done, based on study population, study setting and types of disrespect and abuse on women during the process of childbirth at health facility. Subgroup analysis showed that at community based study prevalence of disrespect and abuse of woman during the process of childbirth at health facilities in Sub-Saharan African was 63.48%((95% CI: 35.22–91.74), whereas the subgroup analysis on facilities based study was 36.89.5%((95% CI: 21.04–52.75) and based on study population health care providers; prevalence of disrespect and abuse of woman during the process of childbirth at health facilities in Sub-Saharan African was 50.01%((95% CI: 27.07–72.95) (Figure S1). On other hand subgroup analysis was done based on types of disrespect and abuse on women during the process of childbirth at health facility: physical violence of women during the process of childbirth at health facilities in Sub-Saharan African from 29 studies was 15.77% (95% CI: 13.38–18.15) (Figure S2), non-confidential care of women during the process of childbirth at health facilities in Sub-Saharan African from 28 studies was 16.87% (95% CI: 14.49–19.24) (Figure S3), abandonment of women during the process of childbirth at health facilities in Sub-Saharan African from 30 studies was 16.86% (95% CI: 13.88–19.84) (Figure S4), and detention of women during the process of childbirth at health facilities in Sub-Saharan African from 22 studies was 4.81% (95% CI: 3.96–5.67) (Figure S5).

## Discussion

Disrespect and abuse of women during the process of childbirth at health facility is violation of the core rights of women, newborns and families. Disrespect and abuse of woman during the process of childbirth at a health facility is a burning issue of an international community. In low and middle income countries due to attention to reducing maternal and child morbidity and mortality by implementing different strategies like increasing institutional delivery and woman friendly care. Meanwhile, disrespect and abuse of woman during the process of childbirth at a health facility is a hurdle to utilize institutional delivery.

Different researches are done on disrespect and abuse of woman during the process of childbirth at health facility across the globe, the findings showed that it is dispersed and inconsistent in the world. The definition of disrespect and abuse of woman during the process of childbirth at health facilities are different terminologies and feelings by health care providers in the world. Bowsers and Hill’s described seven categories of disrespect and abuse of women during the process of childbirth at health facilities are; physical abuse, non-confidential care, non-consented care, non-dignified care, abandonment, discrimination and detention in the facilities [7, 15, 48].

This systemic review and meta-analysis aimed to assess disrespect and abuse of woman during the process of child birth at health facility in Sub-Saharan Africa. Thirty three met the inclusion criteria and included in this systemic review and meta-analysis. The result of this systematic review meta-analysis revealed that the highest (98.0%) prevalence of disrespect and abuse of woman during the process of child birth at health facility was observed a study was done in Nigeria [44] and in Ethiopia 98.9 [27], and the lowest (1.91%) was observed from a study was done in Malawi [33]. This substantial difference between the studies could lack of standardized definitions, instruments and study methods in a study designed disrespect and abuse during the childbirth processing at health facilities introduced the potential error in reporting estimated prevalence, affected generalizability and comparability [49]. Furthermore, this difference might be the women preferred health care providers could be preferred male in some place [50] and others could be preferred female health care providers. Besides, disrespect and abuse as normalized and internalized by both health care providers and women considered as a normal event, the way of data collection like self administration, interview and observation [39], sampling technique, sociocultural difference and way of defining of disrespect and abuse care within the studies. In addition, the ways of health care provider-client approach during childbirth. Some of the health care providers might be made procedures without consent for the wellbeing of women and fetus during childbirth, while due to the communication barrier the women can see as abuse.

Pooled prevalence of disrespect and abuse of woman during the process of childbirth at health facilities was 44.09% in Sub-Saharan Africa. This finding is inconsistent with study were done in India 28% [51], in Brazil 18.3% [52]. The possible explanation might be sociocultural difference, socioeconomic difference, health care provider’s knowledge, attitude and skill difference, the health facilities difference and health system difference, study time, data collection time, sampling technique and the way of defining of disrespect and abuse of woman during the process of childbirth at health facilities within the studies are quite different. This finding is consistent to the study done in Mexico 37.7% [53].

This finding is incomparable with study were done in India 71.3% [12], in Pakistan 97.4% [54], in Pakistan 99.7% [55], and in Peru 97.4% [56]. The possible explanation might be the way of defining of disrespect and abuse, the study population, and the health facility set up difference.

In this meta-analysis non-confidential care (16.87%) and abandonment (16.86%) were highest prevalence of woman during the process of childbirth at health facilities, whereas detention was the lowest prevalence of woman during the process of childbirth at health facilities 4.81%. The possible explanation might be high prevalence of non-confidential care and abandonment of woman during the process of childbirth at health facilities is low number of obstetric care providers and work overload, while low detention is most of sub-Saharan countries have free obstetrics care service.

Implication of this study is synthesis of pooled prevalence of disrespect and abuse woman during the process of child birth at health facilities in Sub-Saharan Africa. Minister of health, relevant stakeholders, international civil society and health care providers to achieve sustainable development goal three to ensure healthy lives and promote wellbeing for all ages and the women have the right to get maximum standard of care during childbirth. The potential limitation of this study were included do not follow the same methodology, various scales, tools, and methods of data collection were carried out. This has an effect on the prevalence of disrespect and abuse during childbirth. The data should be reported with caution, because of the high heterogeneity. Also there is recall bias and limit of language is English. Though, this study synthesis an essential evidence that will help for developing women centered interventions, standardized tool to assess respectful maternity care.

## Conclusion

In this study disrespect and abuse of women during the process of childbirth at health facilities is high compared to other studies, particularly non confidential care and abandonment is high compared to other studies. This study point out that ministry of health, health care providers, maternal health experts shall due attention to women’s right during the process of childbirth at health facilities. The health care providers also should give women centered care and respect the culture of women during maternity care services.

Therefore, Sub-Saharan Africa countries and their policy planners use this information for evidence-based strategy to lessen disrespect and abuse of woman during the process of childbirth at health facilities. This meta-analysis is an input for international community, stakeholders and policy makers to show where we are and to give woman friendly service.

## Supplementary information

**Additional file 1: Table S1.** Quality assessment checklist disrespect and abuse during childbirth and maternity care in Sub-Saharan Africa.

**Additional file 2: Figure S1.** Subgroup based on study population during childbirth and maternity care in Sub-Saharan Africa. **Figure S2.** The forest plot Prevalence of physical abuse during childbirth and maternity care in Sub-Saharan Africa. **Figure S3.** The forest plot Prevalence of non-confidential care during childbirth and maternity care in Sub-Saharan Africa. **Figure S4.** The forest plot Prevalence of abandonment care during childbirth and maternity care in Sub-Saharan Africa. **Figure S5.** The forest plot Prevalence of detention during childbirth and maternity care in Sub-Saharan Africa.

## Data Availability

We do not want to share our data to use for another study.

## Abbreviations

WHO: World health organization
RMC: Respectful maternity care
