# Supplementary material for: Disrespect and abuse of women during the process of childbirth at health facilities in sub-Saharan Africa: a systematic review and meta-analysis
Source: BMC Int Health Hum Rights. 2020 Sep 7;20:23. doi: 10.1186/s12914-020-00242-y (PMC7487593; doi:10.1186/s12914-020-00242-y)
Supplement: Supplementary file 1 — Additional file 1: Table S1. Quality assessment checklist disrespect and abuse during childbirth and maternity care in Sub-Saharan Africa. [file 12914_2020_242_MOESM1_ESM.docx]

Table S1: quality assessment checklist disrespect and abuse during childbirth and maternity care in Sub-Saharan Africa

| Authors | Q1 | Q2 | Q3 | Q4 | Q5 | Q6 | Q7 | Q8 | Q9 | Quality score (9%) |
| --- | --- | --- | --- | --- | --- | --- | --- | --- | --- | --- |
| Asefa and Bekele.,([20](#_ENREF_20)) | Y | N | Y | Y | Y | Y | N | N | N | 5 |
| Anteneh et al., ([21](#_ENREF_21)) | Y | N | Y | Y | Y | Y | N | N | N | 5 |
| Sheferaw et al., ([22](#_ENREF_22)) | Y | N | Y | N | Y | Y | Y | N | Y | 6 |
| *Wasihun B et al.,(*[*23*](#_ENREF_23)*)* | Y | Y | Y | Y | Y | Y | Y | Y | N | 7 |
| Wassihun and Zeleke., ([24](#_ENREF_24)) | Y | Y | Y | Y | Y | Y | N | N | Y | 6 |
| Kathleen P et al., ([25](#_ENREF_25)) | Y | N | Y | Y | Y | Y | N | Y | N | 6 |
| Gebremichael et al., ([26](#_ENREF_26)) | Y | Y | Y | Y | Y | Y | Y | Y | N | 8 |
| Ukke et al.,(27) | Y | N | Y | Y | Y | Y | N | N | N | 5 |
| Mihret.,(28) | Y | N | Y | N | Y | Y | Y | Y | N | 6 |
| Tekle Bobo et al.,(29) | Y | Y | Y | Y | Y | Y | Y | N | Y | 7 |
| Sirajet al.,(30) | Y | Y | Y | N | Y | Y | N | Y | N | 6 |
| *W.Bekele*.,(31) | Y | Y | Y | N | Y | Y | Y | N | Y | 7 |
| Abuya T *et al*.,(32) | Y | Y | Y | Y | Y | Y | N | Y | N | 7 |
| Atai et al.,(33) | Y | Y | Y | Y | N | Y | N | N | N | 5 |
| Sando et al et al., (34)H | Y | N | N | Y | Y | Y | N | Y | N | 5 |
| Sando et al et al., (34)N | Y | Y | Y | Y | Y | Y | Y | Y | N | 8 |
| Margaret E et al., (35) L | Y | N | Y | Y | Y | Y | Y | Y | Y | 9 |
| Margaret E et al., (35) C | Y | N | Y | Y | Y | Y | Y | Y | N | 8 |
| Stephanie K *et al.,(*[*3*](#_ENREF_30)*6)* | Y | Y | Y | Y | Y | Y | Y | Y | Y | 9 |
| Sando et al.,(37) L | Y | Y | N | Y | Y | Y | N | N | N | 5 |
| Sando et al.(37)C | Y | Y | Y | Y | Y | Y | Y | Y | N | 8 |
| Kujawski SA et al.,(38) | Y | Y | Y | Y | Y | Y | Y | Y | Y | 9 |
| Freedman et al., (39) P | Y | Y | Y | Y | Y | Y | N | N | N | 6 |
| Freedman et al., (39)L | Y | Y | Y | N | Y | Y | N | N | N | 6 |
| Elysia et al.,(40) | Y | N | Y | N | Y | N | Y | N | Y | 5 |
| Dunstan R. et al.,(41) | Y | Y | Y | Y | Y | Y | N | Y | N | 7 |
| Galle et al.(42)R | Y | N | N | Y | Y | Y | N | Y | N | 5 |
| Galle et al.(42)U | Y | N | N | Y | Y | Y | N | Y | N | 5 |
| Sethi et al.,(43) | Y | Y | Y | Y | Y | Y | Y | N | Y | 8 |
| Okafor et al., ([4](#_ENREF_34)4) | Y | Y | Y | Y | Y | Y | Y | N | Y | 7 |
| Ijadunola et al.,(45) | Y | Y | N | Y | Y | Y | N | N | N | 5 |
| Moyer et al., (46) | Y | Y | Y | Y | N | Y | N | N | Y | 6 |
| Wesson et al., ([4](#_ENREF_36)7) | Y | Y | N | Y | Y | N | N | N | Y | 5 |

**Key:** **Y**= Yes; **NR**= Not reported

**Question codes:**

1. Was the sample frame appropriate to address the target population?

2. Were study participants sampled in an appropriate way?

3. Was the sample size adequate?

4. Were the study subjects and the setting described in detail?

5. Wasthedataanalysisconductedwithsufficientcoverageoftheidentified sample?

6. Were valid methods used for the identification of the condition?

7. Was the condition measured in a standard, reliable way for all participants?

8. Was there appropriate statistical analysis?

9. was the response rate adequate, and if not, was the low response rate managed appropriately?
