# Supplementary material for: Disrespect and abuse of women during the process of childbirth at health facilities in sub-Saharan Africa: a systematic review and meta-analysis
Source: BMC Int Health Hum Rights. 2020 Sep 7;20:23. doi: 10.1186/s12914-020-00242-y (PMC7487593; doi:10.1186/s12914-020-00242-y)
Supplement: Supplementary file 2 — Additional file 2: Figure S1. Subgroup based on study population during childbirth and maternity care in Sub-Saharan Africa. Figure S2. The forest plot Prevalence of physical abuse during childbirth and maternity care in Sub-Saharan Africa. Figure S3. The forest plot Prevalence of non-confidential care during childbirth and maternity care in Sub-Saharan Africa. Figure S4. The forest plot Prevalence of abandonment care during childbirth and maternity care in Sub-Saharan Africa. Figure S5. The forest plot Prevalence of detention during childbirth and maternity care in Sub-Saharan Africa. [file 12914_2020_242_MOESM2_ESM.docx]

Fig. S1: Subgroup based on study population during childbirth and maternity care in Sub-Saharan Africa

Fig. S2: The forest plot Prevalence of physical abuse during childbirth and maternity care in Sub-Saharan Africa

Fig. S3: The forest plot Prevalence of non-confidential care during childbirth and maternity care in Sub-Saharan Africa

Fig. S4: The forest plot Prevalence of abandonment care during childbirth and maternity care in Sub-Saharan Africa

Fig. S5: The forest plot Prevalence of detention during childbirth and maternity care in Sub-Saharan Africa
